# Lower All-Cause Mortality for Coronary Heart or Stroke Patients Who Adhere Better to Mediterranean Diet-An NHANES Analysis

**DOI:** 10.3390/nu14153203

**Published:** 2022-08-05

**Authors:** Kae-Woei Liang, Chia-Lin Lee, Wei-Ju Liu

**Affiliations:** 1 Cardiovascular Center, Taichung Veterans General Hospital, Taichung 407219, Taiwan; 2 Department of Medicine, School of Medicine, National Yang Ming Chiao Tung University, Taipei 112304, Taiwan; 3 Department of Post-Baccalaureate Medicine, College of Medicine, National Chung Hsing University, Taichung 402204, Taiwan; 4 Department of Medical Research, Taichung Veterans General Hospital, Taichung 407219, Taiwan; 5 Division of Endocrinology and Metabolism, Department of Internal Medicine, Taichung Veterans General Hospital, Taichung 407219, Taiwan; 6 Department of Public Health, College of Public Health, China Medical University, Taichung 406040, Taiwan

**Keywords:** atherosclerotic cardiovascular disease (ASCVD), coronary heart disease (CHD), Mediterranean-style diet (MED), National Health and Nutrition Examination Survey (NHANES), nuts, stroke, whole grains

## Abstract

Consuming a Mediterranean-style diet (MED) is helpful for primary prevention of atherosclerotic cardiovascular disease (ASCVD). However, few studies have compared mortality in ASCVD subjects with different degrees of adherence to the MED diet or have evaluated the contributions of individual diet components. We analyzed National Health and Nutrition Examination Survey (NHANES) participants with a history of coronary heart disease (CHD) or stroke (N = 2052) in a period from 1999 to 2010. Their individual vital status was linked to the National Death Index till the end of 2011. The level of adherence to the MED diet was quantified using a 9-point evaluation score (aMED score). Cox regression models were used to compare the different levels of adherence to the MED diet, and contributions of individual components of the MED diet on cardiovascular, cancer, and all-cause mortality. Among the 2052 subjects with CHD or stroke, 29.0% (596 of 2052) died after a median follow-up of 5.6 years. In Cox regression analysis, higher absolute aMED score (HR 0.798, *p* = 0.0079) or above median aMED score (score 4–9) (HR 0.646, *p* = 0.0013) was negatively associated with all-cause mortality. Among various components of the MED diet, intake of more whole grains or nuts was significantly associated with a lower all-cause mortality. In contrast, a higher aMED score was not associated with less cardiovascular mortality. In a secondary analysis that excluded deaths within 2 years of the NHANES study entry, the above median aMED score (score 4–9) was negatively associated with both all-cause and cardiovascular mortality. In conclusion, subjects with a history of CHD or stroke adhering better to the MED diet in the NHANES study had a lower all-cause mortality during follow-ups. Consuming more whole grains or nuts had a lower all-cause mortality. The protective effect of adherence to the MED diet on decreasing cardiovascular mortality was seen only after excluding those who died within first two years of the NHANES study entry.

## 1. Introduction

The Mediterranean-style diet (MED) is characterized by a high intake of vegetables, legumes, fruits, nuts, cereals, and olive oil, but a low intake of saturated lipids, a moderately high intake of fish, a low-to-moderate intake of dairy products, a low intake of meat and poultry, and a regular but moderate intake of ethanol, primarily in the form of wine typically during meals. Greater adherence to the MED diet, as determined with a score between 0–9, was associated with a significant reduction in total mortality [1] and major adverse cardiovascular events [2] in population-based primary prevention cohorts.

Regarding secondary prevention after having atherosclerotic cardiovascular disease (ASCVD), a MED diet helps in preventing myocardial infarction or cardiovascular death after first myocardial infarction [3]. A MED dietary pattern is associated with a reduced risk of mortality in both men and women with ASCVD after a median 7.7 years of follow-up [4]. In REGARDS participants with existing coronary heart disease (CHD), their MED diet scores are inversely related to hazards of recurrent CHD events and all-cause mortality [5].

The U.S. National Health and Nutrition Examination Survey (NHANES) is a series of nationally representative and cross-sectional studies designed to monitor the health of the American population. Participants are selected from the U.S. non-institutionalized, general population with the design using a complex, stratified, and multi-stage probability-cluster sampling method for in-home interviews and visits at a mobile examination center. Detailed questionnaires regarding dietary components and previous medical history of ASCVD are available [6]. One analysis from the NHANES study showed that lower MED diet scores of the participants are associated with frailty and 8-year mortality risk in adults across all ages [7].

A higher adherence to the MED diet Is known to be useful for primary and secondary prevention of ASCVD and results in a longer survival time [1,2,3,5]. One analysis from a NHANES cohort reported survival benefits of adherence to the MED diet [7]. However, few studies have been undertaken on the association of the MED diet with all-cause, cardiovascular, and cancer mortality in NHANES participants with CHD or stroke. Moreover, no deeper analysis was performed on individual food components of the MED diet for their benefits on mortality. This study aimed to analyze the NHANES participants with a history of ASCVD to evaluate the impact of the MED diet, and contributions of its individual diet components on cardiovascular, cancer, and all-cause mortality.

## 2. Materials and Methods

### 2.1. Study Participants

**Figure 1:**
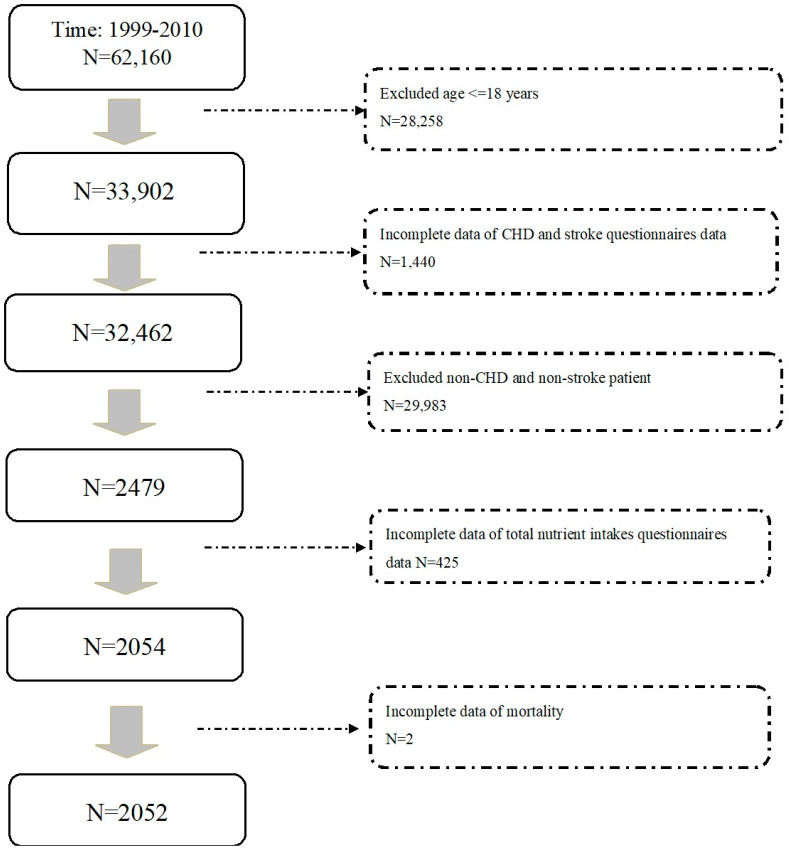
Flow diagram displaying the enrollment of study participants. CHD: coronary heart disease.

NHANES was conducted by the National Center for Health Statistics to assess the health status of community-dwelling individuals in the United States. It contained a series of cross-sectional surveys, which included anthropometric measurements, questionnaires on health and nutritional status, and laboratory tests. Participants in the NHANES had given written informed consent forms. This study was conducted in accordance with the Declaration of Helsinki. Our study protocol has been approved by the Institutional Review Board of Taichung Veterans General Hospital, Taichung, Taiwan (approval number: CE18312A) [6]. Mortality data were linked to the National Death Index through the end of 2011. A flow diagram of the study population is shown in Figure 1. We analyzed participants in the NHANES from 1999 to 2010. Participants excluded from our analyses were those age ≤ 18 years, had missing data from questionnaires for nutrient intakes, incomplete data of CHD or stroke questionnaires, or had incomplete data on vital status. Finally, a total of 2052 subjects with past histories of CHD or stroke were investigated for their dietary consumptions and cardiovascular, cancer, and all-cause mortality (Figure 1). Their median follow-up duration was 5.6 years.

### 2.2. Definition and Scoring for MED Diet

Adherence to the Mediterranean diet was assessed using an alternative Mediterranean diet index (aMED) calculated according to the information in the Food Patterns Equivalents Database (developed by the United States Department of Agriculture; https://www.ars.usda.gov/northeast-areaeltsvillee-md-bhnrceltsvillee-human-nutrition-research-center/food-surveys-research-group/docs/fped-databases/, accessed on 25 January 2021) which was applied to the NHANES [8,9,10]. In summary, the aMED was calculated based on assessed intakes of total fruits, vegetables (except potatoes), whole grains, legumes, nuts, fish, red and processed meat, ratio of monounsaturated to saturated fat, and alcohol [8,9,11]. A single point was assigned to participants whose intakes were greater than the median of the study cohort, except for red and processed meat and alcohol. For alcohol and red/processed meat, one point was assigned to those who had moderate alcohol consumption (10–25 g/day for men and 5–15 g/day for women) or a meat intake that was less than the median for the cohort. If these criteria were not met, participants received point of zero. Hence, the aMED score ranges from 0 to 9, with higher scores representing greater adherence to the Mediterranean diet [8,11,12]. The scores (ranging from 0 to 100) of the Healthy Eating Index 2010 (HEI-2010) scores were used to assess diet quality [13]. The higher these scores, the better was the diet quality (closer conformance to the Dietary Guidelines for Americans).

### 2.3. Mortality Assessment

Mortality data were obtained by linking to the National Death Index up to the end of 2011. The definition of all-cause mortality included all kinds of deaths. The sub-classifications of causes of death into cardiovascular, cancer, respiratory disease, diabetes mellitus, or miscellaneous other causes related mortality were also derived from National Death Index data (coding by International Classification of Diseases).

### 2.4. Statistics

Continuous variables were expressed as a mean (95% confidence interval). Categorical variables were expressed as a number (percentage). The Chi-square test was used to test the statistical difference of categorical variables across the groups. Due to the complexity of the surveys of the NHANES study, the application of conventional estimates was not appropriate. Therefore, all analyses needed to be adequately weighted to represent the U.S. population. We calculated the weighted estimates according to analytic guidelines (National Health and Nutrition Examination Survey: Analytic Guidelines, 2011e2014 and 2015e2016. Available online). We used the analysis of variance (ANOVA) test to examine significant differences in baseline characteristics across different subgroups of dietary patterns. Bonferroni corrections were applied for comparisons among multiple groups. The sample weighted ANOVA test was performed by using a SAS SURVEYREG Procedure according to its User’s Guide. To compare the hazard ratio (HR) and 95% CI for the associations of MED diet and influences of its individual food components on cardiovascular, cancer, and all-cause mortality, we used the weighted Cox proportional hazards regression models of the SURVEYPHREG Procedure with adjustment for relevant variables. The covariables adjusted in Cox regression analysis were age (years), gender (male/female), body mass index (kg/m^2^), race (other races vs. Mexican American), hypertension (with vs. without), and daily energy intake (kcal/day). These variables were picked up because they are important confounding factors in nutritional cohort study and some of them were also significantly different among the aMED score groups. The study participants were compared among a below median aMED score (0–2), median aMED score (3), and above median aMED score (4–9) groups. Their respective associations between the MED diet score and the cardiovascular, cancer, and all-cause mortality were then determined. For avoiding possible contamination of mortality data that were caused by pre-existing severe diseases of the participants, we did a secondary analysis using the Cox proportional hazards regression model excluding deaths in the first two years of the NHANES study entry. Significance was set at a two-sided *p* < 0.05 in all the statistical analyses. We conducted all the statistical analyses using the Statistical Analysis System survey procedures (SAS version 9.4, 2013, Cary, NC, USA).

## 3. Results

### 3.1. Baseline Demographic Data from NHANES Study Participants with CHD or Stroke Related to MED Diet Score

We analyzed a total of 2052 subjects with a history of CHD or stroke in the NHANES database. They were divided according to aMED scores into below median (aMED scores 0–2), median (aMED scores 3), and above median (aMED scores 4–9). Subjects in the above median aMED (scores 4–9) were older, were more male, and had a significantly lower body mass index, serum total cholesterol, and triglyceride. These subjects consumed diets with more fiber, vegetables, whole grains, legumes, seafood, mono-unsaturated fatty acid, and fruits, but less meat, cholesterol, and saturated fatty acids (Table 1).

### 3.2. Cox Regression Analysis of Subjects with a History of CHD or Stroke for All-Cause Mortality

After a median follow-up of 5.6 years, 29.0% (596 of 2052) subjects died. Among those who died (596), 190 of them (31.9%) were due to cardiovascular causes, 99 of them (16.6%) were due to cancer-related deaths, 30 of them (5.0%) due to respiratory diseases, 31 of them (5.2%) due to diabetes-mellitus-related death, and the remaining 246 (41.3%) due to miscellaneous other causes. In the Cox regression analysis, a higher aMED score (score 4–9, above median) was significantly associated with a lower HR 0.646 (*p* = 0.0013) (*p* for trend 0.0023 among the aMED score groups) for all-cause mortality (Table 2). Older age was significantly associated with a higher (HR 1.004, *p* < 0.001) all-cause mortality, whereas female gender (HR 0.790, *p* = 0.0207) and lower food energy intake (HR 0.797, *p* = 0.0369) were associated with lower total mortality. Hypertension was in borderline association with a higher all-cause mortality (HR 1.277, *p* = 0.0752). In another Cox regression analysis, the higher graded absolute value of aMED score (0–9) was also significantly associated with a lower risk (HR 0.798, *p* = 0.0079) for all-cause mortality. Among the individual components of the MED diet, more whole grains or nuts intake were significantly associated with lower all-cause mortality (Table 3). For avoiding possible contamination of mortality data that were caused by pre-existing diseases of the participants, we performed a secondary analysis excluding deaths in the first two years of the NHANES study entry. The results are consistent that a higher aMED score (score 4–9, above median) was significantly associated with a lower all-cause mortality (HR 0.588, *p* = 0.0010) (Table 4).

### 3.3. Cox Regression Analysis of Subjects with a History of CHD or Stroke for Cardiovascular Mortality

Among those who died (596), 190 of them (31.9%) were due to cardiovascular causes. In the Cox regression analysis, aMED score was not significantly associated with cardiovascular mortality (Table 2). In contrast, hypertension (HR 2.208, *p* = 0.0062) and older age (HR 1.004, *p* = 0.0032) were significantly associated with a higher cardiovascular mortality. Female gender (HR 0.509, *p* = 0.0006) and lower food energy intake (HR 0.604, *p* = 0.0128) were associated with lower cardiovascular mortality. Among the individual components of the MED diet, a greater intake of sea food was significantly associated with lower cardiovascular mortality (HR 0.396 (0.163–0.964), *p* = 0.0414) (Table 3) (Supplementary Table S1). For avoiding possible contamination of mortality data that were caused by pre-existing diseases of the participants, we did a secondary analysis excluding deaths within two years of the NHANES study entry. This secondary analysis showed that a higher aMED score (score 4–9, above median) was significantly associated with a lower cardiovascular mortality (HR 0.514, *p* = 0.0323) (Table 4).

### 3.4. Cox Regression Analysis of Subjects with a History of CHD or Stroke for Cancer Mortality

Among those who died (596), 99 of them (16.6%) were due to cancer-related deaths. Greater adherence to the MED diet (aMED score 4–9, above median score) had a borderline significant association (HR 0.543, *p* = 0.0561) for lower cancer mortality (Table 2). Older age (HR 1.003, *p* = 0.0256) was significantly associated with a higher cancer mortality. In contrast, female gender (HR 0.506, *p* = 0.0114) was associated with lower cancer-related mortality. The secondary analysis which excluded deaths in the first two years of the NHANES study entry showed that a higher aMED score (score 4–9, above median) was not significantly associated with a lower cancer mortality (HR 0.690, *p* = 0.2837) (Table 4).

### 3.5. Cox Regression Analysis of Subjects with a History of CHD or Stroke for Respiratory Disease, Diabetes Mellitus Related Death

Among those who died (596), 30 of them (5.0%) due to respiratory diseases, 31 of them (5.2%) due to diabetes mellitus related deaths. In the Cox regression analysis, aMED score was not associated with either respiratory disease or diabetes mellitus related mortality (Table 2).

## 4. Discussions

Better adherence to the MED style diet is known to reduce all-cause mortality and major adverse cardiovascular events in the general population as well as for subjects with ASCVD [1,2,3,4,5]. We have here performed an in-depth study on the impacts of the individual components of the MED style diet on all-cause, cardiovascular, and cancer mortality in relation to the benefits of the MED diet in a cohort with pre-existing ASCVD. The key findings of our study are that subjects with CHD or stroke complying better to the MED diet have a lower all-cause mortality. Among the individual components of the MED diet, more intakes of whole grains or nuts were significantly associated with lower all-cause mortality. The protective effect of better adherence to MED diet on decreasing cardiovascular mortality was seen only after excluding those who died within first two years of the NHANES study entry.

NHANES is a series of nationally representative, cross-sectional studies designed to monitor the health of the U.S. population. One analysis from NHANES participants reported that a lower MED diet score is associated with frailty and 8-year mortality risk in adults across all ages [7]. Another analysis from NHANES showed that better adherence to the MED diet is associated with cardio-protective lipid profiles, glucose metabolism, and inflammation and coagulation levels [10]. Our present study added a new analysis to NHANES participants, focusing on subjects with a history of CHD or stroke, and showed that better adherence to the Mediterranean diet had a lower all-cause mortality.

We also investigated the individual food components of the MED diet regarding their associations with all-cause mortality. Whole grains contain endosperm, germ, and bran, in contrast with refined grains, which have the germ and bran removed during the milling process. Whole grains are good sources of fiber, B vitamins, and trace minerals such as iron, magnesium, and zinc. A meta-analysis reported that higher intake of whole grains is associated with reduced all-cause mortality [14]. Nuts are good sources of dietary fiber, magnesium, polyunsaturated fats, vitamin E, and antioxidants, such as ellagic acid, genistein, resveratrol, and inositol phosphates. Another meta-analysis showed that higher nut intake is associated with reduced all-cause mortality [15]. Our analysis from NHANES participants with ASCVD is in line with the previous reports that greater intakes of whole grains or nuts are significantly associated with lower all-cause mortality (Table 3).

Several previous studies on subjects with ASCVD reported protective effects of the MED diet on the secondary prevention of cardiovascular events or mortality [3,4,5]. However, our present study on participants with pre-existing CHD or stroke, in contrast with their findings, showed no improvement in cardiovascular mortality. One possible explanation of such a discrepancy lies in the definition of CHD or stroke. In the NHAES study, the clinical history of ASCVD was defined based on questionnaires completed by participants themselves, rather than based on medical charts or non-invasive or invasive examinations. Another possible explanation is that in subjects with pre-existing ASCVD, the protective effect from diet is difficult to outweigh the significant protective impacts from medical or interventional treatment. Moreover, the deaths that occurred within 2 years of study entry were probably related with pre-existing severe diseases such as advanced cancer, complex CHD, and heart failure. For avoiding possible contamination of mortality data that were caused by pre-existing diseases, we did a secondary analysis excluding deaths within two years of the NHANES study entry. This secondary analysis showed that a higher aMED score (score 4–9, above median) was significantly associated with a lower cardiovascular mortality (HR 0.514, *p* = 0.0323) (Table 4). A previous study on middle-aged people in Japan reported that a higher intake of fish is associated with substantially reduced risk of coronary heart disease, primarily nonfatal cardiac events [16]. Our analysis from NHANES participants with ASCVD showed that greater intakes of seafood is significantly associated with lower cardiovascular mortality (Table 3) (Supplementary Table S1).

Our analysis showed that subjects with better adherence to MED style diet (aMED score 4–9, above median) had a borderline significant effect (HR 0.543, *p* = 0.0561) in lowering cancer mortality (Table 4). Previous nutritional cohort studies in the general population, or with meta-analysis, have shown better adherence to the MED diet leads to a lower cancer mortality in the follow-up [17,18]. Our study added a new insight that subjects with pre-existing ASCVD who had better adherence to MED style diet trended to have a lower cancer mortality.

There are limitations of this study. First, the definition of a pre-existing history of CHD and stroke was based on questionnaires self-reported by participants but not on hard event records such as myocardial infarction or revascularization history. This could have led to an over-diagnosis of ASCVD. Second, the aMED score was transformed from the dietary pattern reported by participants in questionnaires, the correctness of which was not confirmed by dietitians. Third, the NHANES data collected in 1999 to 2010 might not reflect the most updated nutritional and clinical outcomes in the current practice. Finally, only 190 cardiovascular deaths occurred during follow-up period for the 2052 NHANES participants with preexisting ASCVD. The event rate could be lower than expected to prove the impact of the MED-style diet on cardiovascular mortality in a cohort with pre-existing ASCVD.

## 5. Conclusions

In this study, we found that subjects with a history of CHD or stroke complying more closely to the Mediterranean diet have a lower all-cause mortality during the follow-up period in the NHANES study. Among the individual components of the MED diet, greater intakes of whole grains or nuts are significantly associated with lower all-cause mortality. The protective effect of better adherence to the MED diet on decreasing cardiovascular mortality was seen only after excluding those who died within the first two years of the NHANES study entry. Greater intakes of seafood are significantly associated with lower cardiovascular mortality.

## Figures and Tables

**Table 1:** Baseline demographic data of NHANES participants with coronary heart disease or stroke in alternative Mediterranean diet score.

	Total	aMED	aMED	aMED	*p ^#^* Value	p * Value
		(Below Median)	(Median)	(Above Median)		
		(Score 0–2)	(Score 3)	(Score 4–9)		
Number of subjects	2052	630	511	911		
Age (year)	65.1 (64.2–65.9)	61.2 (59.9–62.4)	64.9 (63.5–66.4)	67.9 (66.7–69.1)	<0.0001	<0.0001
Male, n (%)	1227 (55.2)	361 (50.4)	304 (55.3)	562 (58.5)	0.029	0.0073
BMI (kg/m^2^)	29.5 (29.2–29.8)	30.2 (29.5–30.8)	29.9 (29.3–30.6)	28.8 (28.4–29.3)	<0.0001	<0.0001
SBP (mmHg)	133.8 (132.3–135.3)	133.1 (130.4–135.8)	132.0 (128.7–135.2)	135.4 (133.5–137.3)	<0.0001	<0.0001
DBP (mmHg)	68.4 (67.4–69.4)	70.4 (68.8–72.1)	68.6 (66.9–70.2)	67.0 (65.5–68.4)	<0.0001	<0.0001
Smoking, n (%)	369 (31.7)	181 (46.4)	104 (36.3)	84 (16.8)	<0.0001	<0.0001
HT, n (%)	1469 (69.3)	446 (67.4)	367 (68.2)	656 (71.2)	0.399	0.1614
Cholesterol (mg/dL)	191.5 (188.2–194.9)	194.8 (189.5–200.1)	192.7 (187.6–197.9)	188.6 (184.1–193.1)	<0.0001	<0.0001
HDL-C (mg/dL)	49.1 (48.2–50.0)	48.0 (46.5–49.4)	48.0 (46.5–49.5)	50.4 (49.2–51.7)	<0.0001	<0.0001
TG (mg/dL)	174.4 (166.1–182.8)	178.6 (165.4–191.8)	185.8 (170.6–200.9)	165.1 (154.2–176.0)	<0.0001	<0.0001
FPG (mg/dL)	110.6 (108.0–113.2)	109.0 (104.6–113.4)	114.3 (109.0–119.6)	109.7 (106.2–113.1)	<0.0001	<0.0001
HbA1c, %	6.0 (5.9–6.1)	6.0 (5.8–6.1)	6.1 (6.0–6.3)	6.0 (5.9–6.0)	<0.0001	<0.0001
HOMA-IR	5.2 (4.5–6.0)	5.7 (4.9–6.4)	4.8 (3.9–5.6)	5.2 (3.8–6.6)	<0.0001	<0.0001
eGFR, mL/min/1.73 m^2^	74.3 (72.9–75.7)	78.0 (75.7–80.3)	73.6 (70.8–76.5)	72.1 (70.4–73.8)	<0.0001	<0.0001
**Diet components**						
Calorie intake, kcal/day	1808.9 (1753.4–1864.4)	1700.0 (1596.9–1803.2)	1834.7 (1706.2–1963.2)	1871.2 (1806.4–1935.9)	<0.0001	<0.0001
% from carbohydrate	50.7 (50.1–51.3)	49.0 (47.8–50.2)	50.8 (49.7–51.9)	51.8 (51.2–52.6)	<0.0001	<0.0001
% from fat	33.4 (32.9–34.0)	35.1 (34.1–36.2)	33.2 (32.2–34.1)	32.4 (31.8–33.02)	<0.0001	<0.0001
% from protein	15.9 (15.6–16.1)	15.9 (15.4–16.3)	16.1 (15.6–16.6)	15.8 (15.3–16.2)	<0.0001	<0.0001
HEI 2010 score	50.0 (49.1–50.9)	40.0 (38.9–41.1)	48.3 (47.0–49.5)	58.9 (57.5–60.3)	<0.0001	<0.0001
Fiber (gm)	14.9 (14.3–15.5)	10.1 (9.2–10.9)	14.2 (13.2–15.2)	18.8 (18.0–19.6)	<0.0001	<0.0001
Cholesterol (mg)	258.2 (247.8–268.7)	260.3 (240.9–279.7)	282.2 (251.9–312.5)	243.4 (229.5–257.3)	<0.0001	<0.0001
Calcium (mg)	776.9 (747.7–806.0)	740.6 (679.5–801.7)	749.0 (703.8–794.2)	818.0 (780.9–855.1)	<0.0001	<0.0001
Magnesium (mg)	257.6 (250.1–265.1)	208.2 (195.3–221.1)	249.4 (234.1–264.7)	297.0 (287.6–306.5)	<0.0001	<0.0001
Sodium (mg)	3010.8 (2904.2–3117.4)	2813.2 (2624.4–3002.0)	3149.6 (2918.6–3380.6)	3072.4 (2925.4–3219.4)	<0.0001	<0.0001
Potassium (mg)	2549.0 (2475.6–2622.5)	2196.3 (2054.6–2338.1)	2524.9 (2379.0–2670.9)	2810.9 (2718.0–2903.8)	<0.0001	<0.0001
Saturated fat acids (%)	10.9 (10.7–11.1)	12.8 (12.3–13.3)	10.9 (10.6–11.3)	9.6 (9.3–9.9)	<0.0001	<0.0001
Vegetables (cup)	0.7 (0.6–0.7)	0.4 (0.3–0.4)	0.7 (0.6–0.8)	0.9 (0.8–1.0)	<0.0001	<0.0001
Fruits (cup)	0.9 (0.9–1)	0.4 (0.4–0.5)	0.9 (0.7–1.0)	1.3 (1.2–1.4)	<0.0001	<0.0001
Whole grains (gm)	21.6 (19.6–23.6)	8.8 (7.1–10.5)	19.3 (15.5–23.1)	31.9 (28.6–35.1)	<0.0001	<0.0001
Meat (gm)	70.5 (65.6–75.4)	98.5 (88.0–109.0)	79.3 (68.9–89.7)	46.1 (40.8–51.4)	<0.0001	<0.0001
Legumes (gm)	11.8 (10.0–13.7)	4.2 (2.7–5.7)	9.0 (5.6–12.3)	18.8 (15.4–22.1)	<0.0001	<0.0001
Seafood (gm)	18.5 (14.9–22.1)	5.4 (2.3–8.6)	15.2 (6.8–23.7)	29.4 (23.7–35.0)	<0.0001	<0.0001
Nuts (gm)	13.1 (11.3–14.9)	2.3 (1.3–3.2)	10.6 (7.1–14.1)	22.0 (18.7–25.3)	<0.0001	<0.0001
MUFA/SFA	1.29 (1.17–1.21)	1.01 (0.98–1.04)	1.18 (1.14–1.22)	1.32 (1.29–1.36)	<0.0001	<0.0001
Alcohol (gm)	5.9 (4.8–7.1)	6.3 (4.4–8.2)	4.7 (3.0–6.5)	6.3 (4.3–8.3)	<0.0001	<0.0001

Data are presented as mean (95% confidence interval) or n (%). aMED: Mediterranean-style diet, using a 9-point alternative Mediterranean diet index; BMI: body mass index, DBP: diastolic blood pressure, eGFR, estimated glomerular filtration rate. FPG: fasting plasma glucose, HbA1c: glycated hemoglobin. HDL-C, high-density lipoprotein cholesterol; HEI: healthy eating index, HOMA-IR, homeostasis model assessment-insulin resistance; HT: hypertension, MUFA: monounsaturated fatty acids, NHANES: National Health and Nutrition Examination Survey, SBP: systolic blood pressure, SFA: saturated fatty acids, TG: triglyceride. *p* ^#^: ANOVA test among the three groups. *p* *: post hoc analysis (Bonferroni corrections), comparison between above median (aMED score 4–9) vs. below median (aMED score 0–2).

**Table 2:** Cox regression analysis of NHANES participants with a history of coronary heart disease or stroke for all-cause, cardiovascular, cancer, respiratory disease, diabetes mellitus and all other miscellaneous cause related mortality.

Factors		HR	95% CI of HR		*p* Value
	Death Per 1000 Person-Years		Lower Limit	Upper Limit	
**All-Cause Mortality**					
aMED score (below median, score 0–2)	50.8	1 (reference)			
aMED score (median, score 3)	44.7	0.703	0.527	0.939	0.0175
aMED score (above median, score 4–9)	46.29	0.646	0.498	0.84	0.0013
**Cardiovascular Mortality**					
aMED score (below median, score 0–2)	15.41	1 (reference)			
aMED score (median, score 3)	16.42	0.861	0.480	1.545	0.6121
aMED score (above median, score 4–9)	14.34	0.699	0.429	1.137	0.1470
**Cancer Mortality**					
aMED score (below median, score 0–2)	8.94	1 (reference)			
aMED score (median, score 3)	8.84	0.838	0.394	1.783	0.6432
aMED score (above median, score 4–9)	6.25	0.543	0.290	1.016	0.0561
**Respiratory Diseases Related Mortality**					
aMED score (below median, score 0–2)	3.52	1 (reference)			
aMED score (median, score 3)	1.79	0.385	0.076	1.945	0.2449
aMED score (above median, score 4–9)	2.2	0.519	0.166	1.619	0.2550
**Diabetes Mellitus Related Mortality**					
aMED score (below median, score 0–2)	3.11	1 (reference)			
aMED score (median, score 3)	1.34	0.389	0.079	1.924	0.2439
aMED score (above median, score 4–9)	3.6	0.785	0.236	2.612	0.6900
**All Other Miscellaneous Causes Related Mortality (Residual)**					
aMED score (below median, score 0–2)	13.66	1 (reference)			
aMED score (median, score 3)	12.72	0.806	0.459	1.414	0.4475
aMED score (above median, score 4–9)	15.05	0.785	0.473	1.303	0.3452

aMED: Mediterranean-style diet, using a 9-point alternative Mediterranean diet index. Adjusted for age (years), gender (male/female), body mass index (kg/m^2^), race (other races vs. Mexican American), hypertension (with vs. without), and daily energy intake (kcal/day). CI: confidence interval; HR: hazard ratio.

**Table 3:** Associations of individual food components of the Mediterranean-style diet with all-cause, cardiovascular and cancer mortality in NHANES participants with history of coronary heart disease or stroke.

	All-cause Mortality		CV Mortality		Cancer Mortality	
	Adjusted HR (95% CI) ^a^	*p*	Adjusted HR (95% CI) ^a^	*p*	Adjusted HR (95% CI) ^a^	*p*
Components of aMED						
Alcohol	0.767 (0.516–1.138)	0.1846	0.639 (0.269–1.517)	0.3061	1.239 (0.529–2.901)	0.6178
Red/processed meat	0.995 (0.783–1.264)	0.968	0.969 (0.619–1.517)	0.8887	0.817 (0.455–1.468)	0.4953
Sea food	0.807 (0.597–1.091)	0.1618	0.396 (0.163–0.964)	0.0414	0.892 (0.434–1.832)	0.7532
Whole grains	0.781 (0.615–0.992)	0.0432	0.809 (0.511–1.279)	0.3595	0.619 (0.334–1.144)	0.1241
Legumes	0.979 (0.797–1.202)	0.8386	1.07 (0.684–1.674)	0.7634	0.62 (0.318–1.209)	0.1587
Nuts	0.769 (0.6–0.986)	0.0382	0.756 (0.49–1.166)	0.203	0.948 (0.562–1.599)	0.8393
Fruits	0.916 (0.721–1.164)	0.4701	0.951 (0.609–1.484)	0.8224	0.986 (0.561–1.734)	0.9616
Vegetables	0.793 (0.613–1.027)	0.0777	0.803 (0.54–1.193)	0.2734	0.916 (0.575–1.458)	0.7077
MUFA/SFA	0.923 (0.733–1.163)	0.4923	0.868 (0.576–1.31)	0.4974	0.808 (0.487–1.341)	0.4054

aMED: alternative Mediterranean Diet Index; CI: confidence interval; CV: cardiovascular; HR: hazard ratio; MUFA: monounsaturated fatty acid; SFA: saturated fatty acid. ^a^ Adjusted for age (years), gender (male/female), body mass index (kg/m^2^), race (other races vs. Mexican American), hypertension (with vs. without), and daily energy intake (kcal/day).

**Table 4:** Secondary analysis using Cox regression in NHANES participants with a history of coronary heart disease or stroke for all-cause, cardiovascular, and cancer mortality that excluded those who died within the first two years of the NHANES study entry.

Factors	Deaths Per 1000	HR	95% CI of HR		*p* Value
	Person-Years		Lower Limit	Upper Limit	
**All-Cause Mortality**					
aMED score (below median, score 0–2)	40.42	1 (reference)			
aMED score (median, score 3)	29.53	0.571	0.393	0.829	0.0037
aMED score (above median, score 4–9)	36.09	0.588	0.431	0.801	0.0010
**Cardiovascular Mortality**					
aMED score (below median, score 0–2)	11.76	1 (reference)			
aMED score (median, score 3)	8.47	0.586	0.277	1.239	0.1596
aMED score (above median, score 4–9)	10.28	0.514	0.280	0.944	0.0323
**Cancer Mortality**					
aMED score (below median, score 0–2)	6.13	1 (reference)			
aMED score (median, score 3)	7.63	0.986	0.407	2.393	0.9754
aMED score (above median, score 4–9)	5.52	0.690	0.347	1.368	0.2837

aMED: Mediterranean-style diet, using a 9-point alternative Mediterranean diet index. CI: confidence interval; HR: hazard ratio. Adjusted for age (years), gender (male/female), body mass index (kg/m^2^), race (other races vs. Mexican American), hypertension (with vs. without), and daily energy intake (kcal/day).

## Data Availability

The datasets generated and/or analyzed during the current study are available in the National Center for Health Statistics, Centers for Disease Control and Prevention (https://wwwn.cdc.gov/nchs/nhanes/Search/DataPage.aspx?Component=Dietary&CycleBeginYear=2005, accessed on 25 January 2021).

## Supplementary Materials

The following are available online at https://www.mdpi.com/article/10.3390/nu14153203/s1, Table S1: The Mediterranean diet food components with their respective adherence data to all-cause, cardiovascular and cancer mortality.
