# Supplementary material for: Lower All-Cause Mortality for Coronary Heart or Stroke Patients Who Adhere Better to Mediterranean Diet-An NHANES Analysis
Source: Nutrients. 2022 Aug 5;14(15):3203. doi: 10.3390/nu14153203 (PMC9370677; doi:10.3390/nu14153203)
Supplement: Supplementary file 1 [file nutrients-14-03203-s001.zip › nutrients-1790657-supplementary.pdf]

nutrients

MDPI

Supplementary Materials

Lower All-Cause Mortality for Coronary Heart or Stroke Patients Who Adhere Better to Mediterranean Diet-An NHANES Analysis

Kae-Woei Liang [1,2,3,*], Chia-Lin Lee [2,3,4,5,6] and Wei-Ju Liu [4]

Table S1. The Mediterranean diet food components with their respective adherence data to all-cause, cardiovascular and cancer mortality.

Mortality	N	All-cause mortality	CV mortality	Cancer mortality
Alcohol (0)	1928	565 (26.45)	179 (8.62)	91 (4.16)
Alcohol (1)	124	31 (22.01)	11 (6.28)	8 (6.85)
missing	0	0	6	6
Red/processed meat (0)	1011	289 (25.58)	93 (7.95)	57 (4.85)
Red/processed meat (1)	1041	307 (26.77)	97 (9.01)	42 (3.8)
missing	0	0	6	6
Sea food (0)	1707	515 (27.27)	170 (9.24)	86 (4.51)
Sea food (1)	345	81 (21.08)	20 (4.93)	13 (3.46)
missing	0	0	6	6
Whole grains (0)	1034	299 (26.06)	93 (8.44)	58 (4.83)
Whole grains (1)	1018	297 (26.29)	97 (8.52)	41 (3.82)
missing	0	0	6	6
Legumes (0)	1450	402 (25.04)	130 (8.34)	71 (4.53)
Legumes (1)	602	194 (28.97)	60 (8.83)	28 (3.83)
missing	0	0	6	6
Nuts (0)	1417	419 (27.18)	133 (9)	68 (4.37)
Nuts (1)	635	177 (24.26)	57 (7.48)	31 (4.24)
missing	0	0	6	6
Fruits (0)	1005	250 (22.86)	90 (7.69)	42 (3.63)
Fruits (1)	1047	346 (29.49)	100 (9.27)	57 (5.02)
missing	0	0	6	6
Vegetables (0)	1055	333 (29.43)	106 (9.35)	57 (4.9)
Vegetables (1)	997	263 (22.92)	84 (7.61)	42 (3.76)
missing	0	0	6	6
MUFA/SFA (0)	978	282 (25.16)	98 (8.63)	50 (4.45)
MUFA/SFA (1)	1074	314 (27.2)	92 (8.33)	49 (4.21)
missing	0	0	7	7

CV: cardiovascular; MUFA: monounsaturated fatty acid; SFA: saturated fatty acid.
Food component (0): alternative Mediterranean Diet Index (aMED) score gets 0.
Food component (1): aMED score gets 1.
Mortality data: N (weighted mortality rate %).
